# Differential gene expression between functionally specialized polyps of the colonial hydrozoan *Hydractinia symbiolongicarpus* (Phylum Cnidaria)

**DOI:** 10.1186/1471-2164-15-406

**Published:** 2014-05-28

**Authors:** Steven M Sanders, Mariya Shcheglovitova, Paulyn Cartwright

**Affiliations:** Department of Ecology and Evolutionary Biology, University of Kansas, Lawrence, Kansas 66045 USA; Department of Biological Sciences, George Washington University, Washington, DC 20052 USA

**Keywords:** *Hydractinia symbiolongicarpus*, RNA-Seq, Polymorphism, Differential expression, Transcriptome assembly, Annotation

## Abstract

**Background:**

A colony of the hydrozoan *Hydractinia symbiolongicarpus* comprises genetically identical yet morphologically distinct and functionally specialized polyp types. The main labor divisions are between feeding, reproduction and defense. In *H. symbiolongicarpus*, the feeding polyp (called a gastrozooid) has elongated tentacles and a mouth, which are absent in the reproductive polyp (gonozooid) and defensive polyp (dactylozooid). Instead, the dactylozooid has an extended body column with an abundance of stinging cells (nematocysts) and the gonozooid bears gonophores on its body column. Morphological differences between polyp types can be attributed to simple changes in their axial patterning during development, and it has long been hypothesized that these specialized polyps arose through evolutionary alterations in oral-aboral patterning of the ancestral gastrozooid.

**Results:**

An assembly of 66,508 transcripts (>200 bp) were generated using short-read Illumina RNA-Seq libraries constructed from feeding, reproductive, and defensive polyps of *H. symbiolongicarpus*. Using several different annotation methods, approximately 54% of the transcripts were annotated. Differential expression analyses were conducted between these three polyp types to isolate genes that may be involved in functional, histological, and pattering differences between polyp types. Nearly 7 K transcripts were differentially expressed in a polyp-specific manner, including members of the homeodomain, myosin, toxin and BMP gene families. We report the spatial expression of a subset of these polyp-specific transcripts to validate our differential expression analyses.

**Conclusions:**

While potentially originating through simple changes in patterning, polymorphic polyps in *Hydractinia* are the result of differentially expressed functional, structural, and patterning genes.

The differentially expressed genes identified in our study provide a starting point for future investigations of the developmental patterning and functional differences that are displayed in the different polyp types that confer a division of labor within a colony of *H. symbiolongicarpus*.

**Electronic supplementary material:**

The online version of this article (doi:10.1186/1471-2164-15-406) contains supplementary material, which is available to authorized users.

## Background

Colonial hydrozoans are composed of individual polyps connected through continuous epithelia and a shared gastrovascular cavity. Hydrozoans are members of the phylum Cnidaria, which are characterized by their diploblastic construction, comprising only two epithelial layers, the epidermis and gastrodermis. Despite their simple epithelial construction, many hydrozoan species evolved complex colonies through functional specialization of genetically identical yet morphologically distinct polyp types, conferring a division of labor within the colony [1–3]. This division of labor is known as polyp polymorphism [1–3].

**Figure 1:**
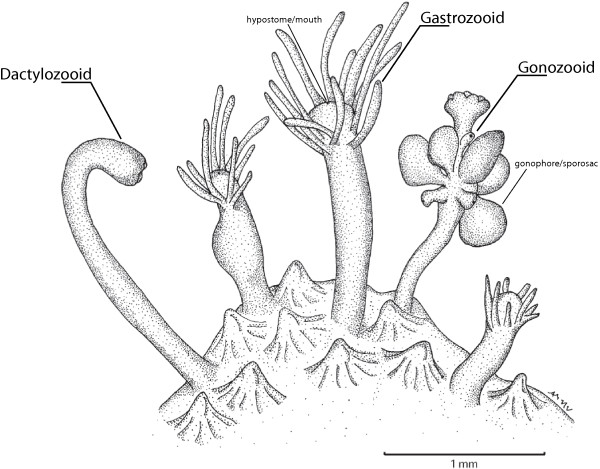
**Colony of** ***Hydractinia symbiolongicarpus.*** Illustration showing the different polymorphic polyps that comprise the *H. symbiolongicarpus* colony. Modified from Cartwright & Nawrocki [4].

The main labor divisions are between feeding, reproduction, and defense, where specialized polyp types are morphologically distinct, reflecting their particular functions. *Hydractinia symbiolongicarpus* has four different polyp types (Figure 1). The feeding polyp (called a gastrozooid) has a mouth and tentacles, which are absent in the reproductive polyp (gonozooid), defensive and food gathering polyp (dactylozooid), and the less common defensive polyp (tentaculozooid, not shown). The dactylozooid has an elongated body column with an abundance of epithelial muscular cells and nematocytes (stinging cells). The gonozooid bears gonophores, which house the gametes. The gonozooid and dactylozooid are similar in their distal ends, with clusters of nematocysts and lacking a functional mouth and elongate tentacles. The tentaculozooid resembles a tentacle of the gastrozooid, but is the size of an individual polyp.

It has long been hypothesized that these specialized polyps arose through evolutionary alterations in oral-aboral patterning in the ancestral gastrozooid [5–7]. Previous studies using candidate gene approaches have identified patterning genes specific to different polyp types. Cartwright et al. [8] focused on the involvement of *Cnox-2*, a parahox gene, in patterning these different polyp types of *H. symbiolongicarpus*. Immunolocalization of the *Cnox-2* protein showed expression in body column tissue and down-regulation in oral structures of the gastrozooid. Mokady et al. [9] compared expression of *Cn-ems* (*empty spiracles* homolog) between gastrozooids and gonozooids of *H. symbiolongicarpus*. Whole mount *in situ* hybridization revealed no expression of *Cn-ems* in the gonozooid, while mRNAs were detected in the gastrodermal epithelia (“digestive cells”) of the gastrozooid.

More recently, Siebert et al. [10] used an RNA-Seq approach to examine differential expression between several polyp types of another hydrozoan, the siphonophore *Nanomia bijuga*. Although the focus of their paper was to evaluate next generation sequencing (NGS) platforms for differential expression (DE), they confirmed, through whole mount *in situ* hybridization, that at least one gene identified through their DE analyses (*isogroup03256*) was expressed in a polyp specific manner.

With the advent of NGS technologies, an unbiased approach to identify genes involved in the differentiation of different tissues (e.g. [11]) and developmental stages (e.g. [12]), or as well as those that are differentially expressed between species (e.g. [13]) can be made without reference to particular candidate genes. We report a transcriptome assembly, annotations, and DE analyses between three different polyp types in *H. symbiolongicarpus.* Our results, confirmed, with whole mount *in situ* hybridization, that DE analyses using RNA-Seq is a powerful approach for identifying genes and pathways involved in conferring a division of labor within this colonial organism.

## Results and discussion

### Transcriptome assembly and annotation

**Figure 2:**
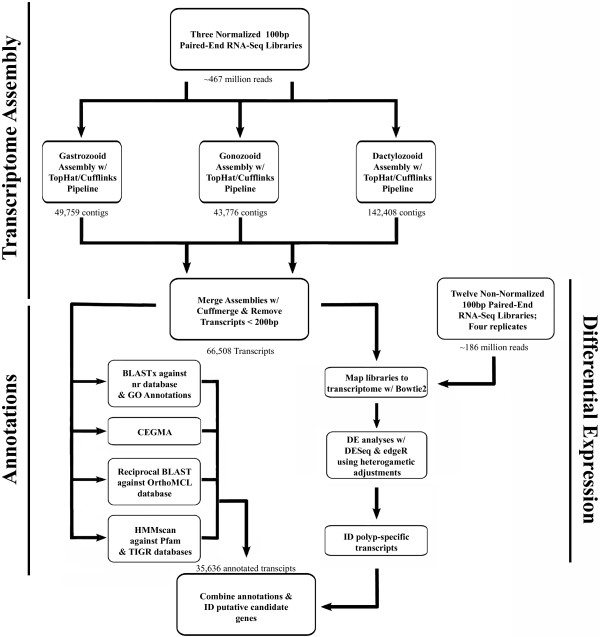
**Workflow of transcriptome assembly through annotation and differential expression analyses.** Raw reads from three normalized libraries were filtered based on quality score and separately mapped to unpublished genomic scaffolds of *H. symbiolongicarpus* using TopHat 2.0.6, assembled using Cufflinks 2.1.1, merged into a single assembly using cuffmerge, and filtered by transcript size, removing assembled transcripts less than 200 bp in length. Blast2GO, CEGMA, HMMscan, and orthoMCL were used to annotate the transcriptome. Differential expression began with mapping 12 non-normalized libraries to the final transcriptome assembly with Bowtie2. DE was then assessed with DESeq and edgeR and polyp-specific DEs were compared to the annotated transcriptome.

From the three normalized libraries, 49,759, 43,776, and 142,408 contigs were assembled for the gastrozooid, gonozooid, and dactylozooid, respectively. Individual transcriptomes were merged into a single assembly of 101,518 unique transcripts using cuffmerge (Figure 2). Cuffmerge merges novel and common transcripts into a single assembly and removes artifact constructions, improving the overall quality of the assembly. This step allows for easy annotation and differential expression analyses of a single assembly, without concerns regarding orthology assignments between multiple assemblies. After filtering for transcripts less than 200 bp in length, our final assembly consisted of 66,508 transcripts, with an N50 of 1,451 bp (Additional file 1).

**Figure 3:**
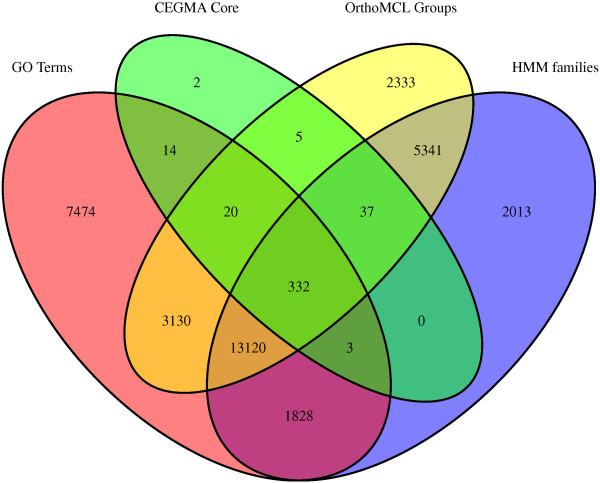
**Venn diagram displaying the number of transcripts annotated by each method.** Gene Ontology terms were added with Blast2GO using the BLASTX algorithm against NCBI’s nr protein database and a threshold of 1 × 10^-03^. A set of conserved eukaryotic genes was identified with CEGMA. HMM protein families from the PFAM and TIGR databases were assigned to the amino acid translation of the most likely reading frame for each transcript (identified using an open reading frame prediction tool) using HMMscan under default settings. HMMscan annotations were constrained to a significance threshold of 0.01. Orthogroups were assigned to the same amino acid translations using the orthoMCL web server.

Approximately 54% of the transcriptome (35,636 transcripts) was annotated using Blast2GO, CEGMA, orthoMCL, and HMMscan (Additional file 2), with these transcripts showing significant similarity to sequences in at least one database in our annotation pipeline (Figures 2 and 3). These include 416 (91%) of the “core” and 238 (96%) of the “ultra-conserved” eukaryotic genes identified using CEGMA (Additional file 3). Figure 3 shows the number of transcripts annotated by one or more of the annotation methods.

### Differential expression analyses

**Figure 4:**
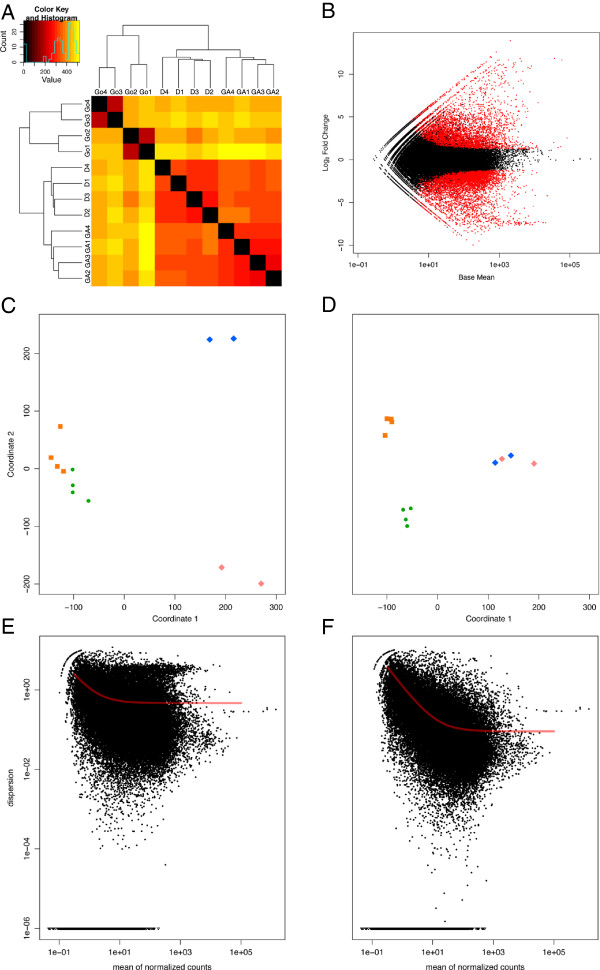
**Effects of heterogametic expression on library distances. A**. Heatmap of the Euclidean distances between all twelve libraries prior to heterogametic adjustments. Samples Go1 and Go2 correspond to female gonozooid libraries, while Go3 and Go4 correspond to male gonozooid libraries. **B**. MA plot of the DE analysis between male and female gonozooid libraries in DESeq. Red dots indicate statistically significant DE transcripts. Log_2_FoldChange > 0 corresponds to expression levels higher in the male gonozooid libraries, and Log_2_FoldChange < 0 corresponds to expression levels higher in the female gonozooid libraries. **C**. Euclidean distances plotted in two dimensions prior to heterogametic adjustments. (Legend: orange square-gastrozooids; green circle-dactylozooids; blue diamond-male gonozooids; pink diamond-female gonozooids). **D**. Euclidean distances plotted in two dimensions after all statistically significant heterogametic transcripts are removed. **E**. Plot of the estimated dispersion values against the mean of normalized counts of each transcript when binning both male and female gonozooid libraries in a single condition. Fitted dispersion values indicated by the red line. **F**. Plot of the estimated dispersion values against the mean of normalized counts of each transcript when male and female gonozooid libraries treated as separate conditions.

**Figure 5:**
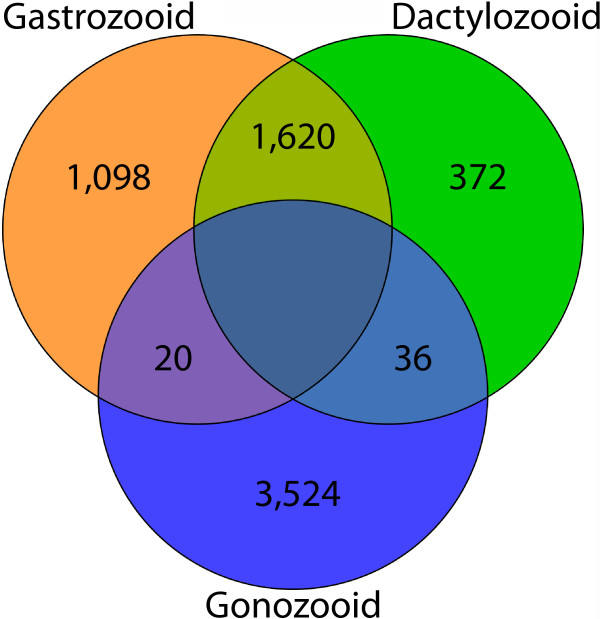
**Venn diagram showing numbers of polyp-specific transcripts.** Transcripts significantly up- or down-regulated (p_adj_ < 0.05) in a particular polyp when compared to either of the other two polyp types from both edgeR and DESeq were considered polyp-specific. The intersection of each circle is the number of transcripts down-regulated in the polyp type excluded from that intersection (e.g. 20 transcripts are down-regulated in the dactylozooids). Down-regulation of a transcript in a particular polyp type equates to equivocal up-regulated expression in the other two polyp types.

Statistically significant differences in expression between different polyp types were detected using two DE packages, DESeq and edgeR. Figure 4C and D shows the effect of heterogametic adjustments on the Euclidean distances (sum of the pairwise distance across all transcripts) between libraries. Both DESeq and edgeR reveal that dactylozooids and gastrozooids share the fewest number of DE transcripts and the smallest change in the number of DEs recovered after the heterogametic adjustments (Table 1), while DE analyses including gonozooids show a much larger increase in the number of DE transcripts after those adjustments. This large increase can be explained by the huge amount of variability found when ignoring the sexual differences between gametic tissues in gonozooid samples. The DE analysis between the male and female gonozooid libraries identified 11,798 (DESeq) and 12,886 (edgeR) transcripts significantly up- or down-regulated (Table 1, Figure 4B). Removal of all male/female DE transcripts clusters gonozooid samples by polyp type rather than sex and increases the distance between gastrozooids and dactylozooids (Figure 4D), while treating male and female gonozooid libraries as different conditions reduces the average dispersion estimate for each transcript, essentially increasing the power of the DE analyses (Figure 4E and F). Yet, even after the heterogametic adjustments, gonozooids still have the largest number of polyp-specific transcripts (transcripts that are strictly up- or down-regulated in a particular polyp type when compared to other polyps; Figure 5, Table 2, Additional file 4).

**Table 1:** **Number of DE transcripts in different pairwise comparisons of the libraries**

	Full dataset		Adjusted dataset	
	DESeq	edgeR	DESeq	edgeR
**Dact vs Gast**	662	2,498	2,062	4,230
**Dact vs Gono**	2,312	16,879	10,341	18,899
**Gast vs Gono**	4,245	16,889	11,908	18,744
**Male vs Female**	11,798	12,886		

Full Dataset corresponds to the number of transcripts recovered from DE analyses (p_adj_ < .05). Adjusted dataset refers to counts following heterogametic adjustments.

**Table 2:** **Number of transcripts identified as always up- or down-regulated in a specific polyp**

	padj < .05					
	DESeq		edgeR		Both	
	Up	Down	Up	Down	Up	Down
**Gastrozooid**	1,067	40	1,934	148	955	31
**Gonozooid**	3,505	3,405	11,304	1,851	3,491	1,562
**Dactylozooid**	444	29	999	180	332	20

Transcripts that have support as polyp-specific (must be significant in only two of the three pairwise comparisons) by both DESeq and edgeR are what we refer to as polyp-specific in the text.

Our DE analyses revealed several polyp-specific genes that are consistent with previous studies using candidate gene approaches in cnidarians as discussed below. Furthermore, our analyses revealed additional genes that were not previously considered to play specific developmental, functional and/or structural roles in cnidarians. Below we summarize of few of these results and suggest areas of interest for further study.

### Gametogenic expression

While adjusting for differences in gene expression between males and females greatly reduced the effect of heterogametic expression on the DE analyses, genes likely involved in non-sex specific gametogenesis were found up-regulated in the gonozooids. Of the 76 polyp-specific DE transcripts annotated with functional terms that include mitosis, cell cycle, and germline maintenance (Additional file 5), 69 are up-regulated in the gonozooids, including four DE transcripts annotated as known hydrozoan stem cell markers *nanos*[14]*, vasa*[15], and *piwi*[16]. This is consistent with expression studies of *nanos* and *vasa* genes in a closely related species, *Hydractinia echinata*[14, 15].

### Homeobox genes

Several homeobox transcripts are differentially expressed between different polyp types (Additional file 5). Homeobox genes up-regulated in the gastrozooid include members of the LIM (*lhx*), sine oculus (*six*), empty spiracles (*ems*), and PRD classes, confirmed by molecular phylogenetic analysis of cnidarian homeodomains (Additional file 5; Additional file 6). The up-regulation of the empty spiracles homolog (100% bootstrap [BS] support; Additional file 5; Additional file 6), *Cn-ems*, is consistent with the findings of Mokady et al. [9] discussed previously*.* Up-regulated gastrozooid expression of two *lhx*-like transcripts, one *six*-like and one *orthopedia* (PRD class) transcript (100%, 99%, and 98% BS support, respectively; Additional file 5; Additional file 6) is also consistent with expression studies in other cnidarians, including *Aurelia*[17], *Nematostella*[18–20], *Craspedacusta*[21], *Cladonema*[22], and *Podocoryna*[22], where their expression was found in regions specific to feeding and/or digestion, including tentacles and gastric tissue.

One of the homeodomain-containing transcripts up-regulated in the gonozooid belongs to the POU class (Additional file 5; Additional file 6). Expression of POU homeodomain transcription factors has also been categorized in other cnidarians, including *Aurelia*[23] and *H. echinata*[24]. In *H. echinata*, the POU gene, *pln*, is expressed around interstitial stem cell (i-cells) [24]. The *H. symbiolongicarpus* ortholog to *pln* (100% BS support; Additional file 5; Additional file 6) is up-regulated in the gonozooid, which is consistent with that of the other stem cell markers mentioned previously.

### Myosins

Myosin genes are a superfamily of molecular motor proteins, primarily associated with muscular contraction and cell movement. Here we find a complex pattern of differential expression of several different myosin transcripts up-regulated in each polyp type (four, six, and four transcripts in the gastrozooids, gonozooids, and dactylozooids, respectively), spanning several myosin classes (Additional file 5, Additional file 7). Of particular note is the up-regulation of a tropomyosin transcript in the gonozooids. In the hydrozoan *Podocoryna carnea*, a tropomyosin, *tpm2*, is expressed solely in the striated muscle of the developing and adult medusa life cycle stages and not in the polyp [25], as opposed to *tpm1*, which is ubiquitously expressed in both polyp and medusae stages [26]. In *Hydractinia*, gonophore development is greatly truncated and never reaches the medusae stage. Instead *Hydractinia* forms sporosacs, which are believed to lack all medusae like features, including striated muscle necessary for medusae to swim [27–29]. Phylogenetic analysis of cnidarian myosins did not recover any well-supported orthologous relationship between this polyp-specific tropomyosin and other known cnidarian tropomyosins, although orthology assignments of several other polyp-specific myosins were revealed (Additional file 7). Further discovery of tropomyosin genes in additional cnidarian taxa are necessary to determine if different tropomyosin orthologs are specific to certain medusae features and/or reduced developmental forms.

### Toxins

While research into the characterization and properties of cnidarian toxins is on the rise, very little is known of their function and location of endogenous expression [30]. We identified 13 DE transcripts annotated as some type of toxin (three up-regulated in the gastrozooids, seven in dactylozooids, and one in gonozooids; two down-regulated in gonozooids; Additional file 5). Phylogenetic analysis of cnidarian toxins recovered a monophyletic cluster of six *H. symbiolongicarpus* ‘echotoxin’ transcripts as sister to a group of anthozoan toxin genes (60% BS support; Additional file 8), and a strongly supported (92% BS; Additional file 8) sister relationship between a four *H. symbiolongicarpus* toxins and two scyphozoan toxins from *Aurelia* (TX1 and TX2; Additional file 8). The remaining three polyp-specific toxins were not placed in any well-supported orthologous groups. Further study is warranted to determine if these toxins each play a unique role in different functions, such as prey capture, defense, and/or digestion.

### Astacins

A large number of transcripts belonging to the astacin subfamily are up-regulated in the gastrozooid (44 total, Additional file 5; Additional file 9), consistent with one of their roles as digestive enzymes in other metazoans [31–34]. Expression studies of several astacin genes in hydrozoans also suggest a role in digestion. In *P. carnea, pmp1* is expressed in both the mouth of the polyp and the manubrium of the medusa stage [35]. Immunolocalization of the HMP1 protein found it expressed in the head and tentacle regions of *Hydra*[36], while Kumpfmüller et al. [37] found *farm1* expressed in both the epi and gastrodermal layers of gastric region of *Hydra*.

Its important to note that digestion is just one function of the astacin subfamily. Another function is in regeneration, as shown in *H. echinata*, where Möhrlen et al. [38] found astacins *hea1* and *hea2* expressed throughout development and soon after the gastrozooid is subjected to tissue injury (expression in other polyp types not mentioned). HMP1 was also up-regulated during head regeneration in *Hydra*[36]. Orthologs of *hea1* and *hea2* were among the 44 gastrozooid-specific astacins in our study (94% and 99% BS support, respectively; see Additional file 5; Additional file 9). Up-regulation of these transcripts may be a result of tissue damage response during dissections prior to RNA extractions. However, it is interesting that they are specific to the gastrozooid, suggesting that gonozooids and dactylozooids may have different regenerative properties than gastrozooids [1, 3, 39].

### *In situ* hybridization

**Figure 6:**
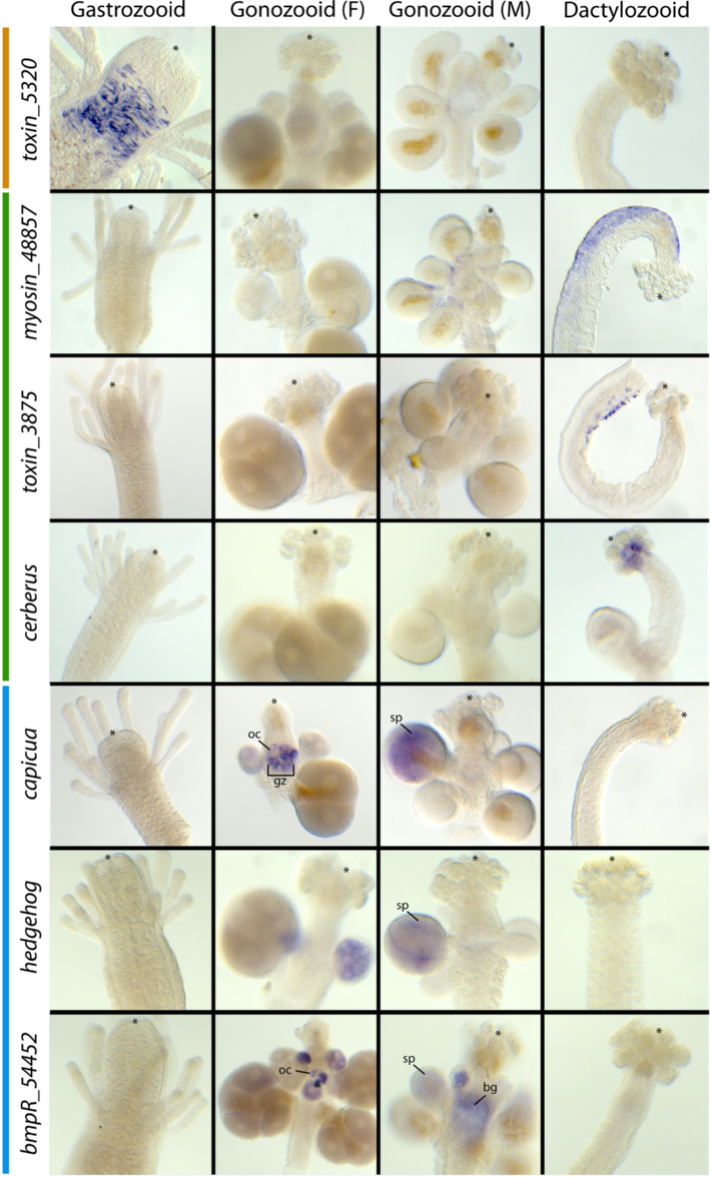
**Images of whole mount in situ hybridization of polyp-specific transcripts.** * = distal end of polyp; oc = oocytes; gz = germinal zone; sp = sperm; bg = non-specific staining.

Figure 6 shows whole mount *in situ* hybridization (ISH) results of several polyp-specific transcripts identified through the DE analyses (listed in Table 3). DE analyses reported several different toxin transcripts to be differentially expressed between the different polyps. Polyp specificity of one of the three toxins identified as gastrozooid-specific by DE analyses, referred to here as *toxin_5320,* was confirmed by ISH. This transcript was expressed solely in specific gastrodermal cells around the base of the hypostome/tentacle margin of the gastrozooids. Three distinct cell types populate the gastrodermis of the hypostome in *Hydractinia*: gastrodermal epithelia (including digestive cells) and two glandular cell types (spumeous and spherulous cells) [40–42]. *Toxin_5320* expression appears to be limited to the spherulous cells of the hypostome (Figure 6, Additional file 10). DE analyses found *toxin_3875* to be dactylozooid-specific and ISH found expression to be limited to nematocytes primarily found in the proximal portion of the body column of the dactylozooid (Figure 6, Additional file 10).

**Table 3:** **Polyp-specific DE transcripts analyzed with whole mount in situ hybridization**

Transcript ID	Name	Top blast hit	HMM family	Polyp type
Hs_transcript_5320	toxin_5320	echotoxin a	Sea anemone cytotoxic protein	Gastrozooid
Hs_transcript_48857	myosin_48857	myosin heavy chain isoform a	Myosin tail	Dactylozooid
Hs_transcript_3875	toxin_3875	echotoxin a	Sea anemone cytotoxic protein	Dactylozooid
Hs_transcript_44185	cerberus	cerberus 1	DAN domain	Dactylozooid
Hs_transcript_16185	capicua	transcription factor capicua	HMG (high mobility group)	Gonozooid
Hs_transcript_1524	hedgehog	indian hedgehog b	Hint module	Gonozooid
Hs_transcript_54452	bmpR_54452	BMP receptor	Protein kinase domain	Gonozooid

A myosin gene, referred to here as *myosin_48857*, was identified as a dactylozooid-specific gene by DE analyses. ISH confirmed this, recovering expression limited to the ectoderm of the body column of the extended side when the dactylozooid is curled in on itself (Figure 6). Minor expression is also detected around the base of the gonophores and on the body column of some gonozooids (not shown).

The gene *cerberus* is also found to be dactylozooid-specific by DE analysis. This gene is only expressed in the gastrodermis beneath the clusters of nematocysts at the distal end of the dactylozooid (Figure 6). Expression studies of *cerberus* in other metazoans have shown it to act as an antagonist of TGF-ß and Wnt signaling [43, 44]. Here, expression in the dactylozooids is consistent with its antagonist role in Wnt signaling. *H. symbiolongicarpus*’ canonical wnt, *HsWnt3* [GenBank:KF745052], is expressed at the distal tip of the dactylozooid (not shown, unpublished). *Cerberus* is expressed at the proximal boundary of *HsWnt3* expression, potentially acting to maintain *HsWnt3*’s expression boundary. This however appears to be specific to the dactylozooids, as *H. symbiolongicarpus* feeding polyps express Wnt3 (not shown, unpublished), similar to other hydrozoan feeding polyps including *H. echinata*[45–47], *P. carnea* (unpublished), and *Hydra*[48–52]), but do not express *cerberus* (Figure 6).

ISH also confirmed the specificity of several gonozooid-specific DE transcripts. Expression of the *hedgehog* homolog is restricted to the gastrodermis of both male and female gonophores (Figure 6). Expression of a bmp receptor gene, tentatively called *bmpR_54452,* and *capicua* are primarily limited to developing oocytes in females and the gastrodermis of male gonophores (Figure 6). ISH expression patterns of these transcripts suggest their involvement in some stage of meiotic/mitotic division during gametogenesis. In *Hydractinia*, oogenesis begins in the germinal zone (body column) of female gonozooids and oocyte differentiation continues after moving into the gonophores [1, 2, 53], while spermatogenesis takes place entirely in the gastrodermis of the male gonophores [2, 53].

For several of these transcripts, expression in the females might not only be associated with germline proliferation, but with maternal transcript generation as well. Maternal expression of *capicua* and BMP receptors in early embryonic development has been reported in other metazoans [54, 55]*.* Expression corresponding to maternal transcript generation is consistent with strong expression around developing oocytes in the germinal zone. By contrast, ISH of *hedgehog* in *Hydractinia* recovered no expression in the germinal zone of female gonophores (Figure 6). Instead, its expression was limited to the gastrodermal tissues surrounding maturing oocytes in female gonophores. This is consistent with *hedgehog* genes implicated in germline proliferation and differentiation in other metazoans [56, 57] and in *Nematostella,* where one *hedgehog* appears to be involved in germline proliferation, but lacks maternal expression [58].

## Conclusions

Our non-biased approach of characterizing differential expression in different polyp types enabled us to identify key genes potentially involved in the morphological and functional differences between these different polyps. However, in interpreting results from a DE analysis, it is important to understand the distinction between biological relevance and statistical significance. We do not propose that every transcript in our list of putative polyp-specific genes is involved in the patterning or function of these different polyps, nor do we report to have captured all polyp-specific genes. One type of information not captured in this method would be those genes whose spatial or temporal expression (but not abundance) confers differences between polyp types. For example, the parahox gene *Cnox-2,* which was shown to be expressed in all polyps uniformly except for the oral region of the gastrozooid [8] was not recovered in the DE analysis. This is likely due to the fact that *Cnox-2* has different patterns of expression but not distinct differences in abundance between polyp types.

Even given the potential limitations, this unbiased approach of RNA-Seq DE analysis, validated through *in situ* hybridization, identified many potential patterning and functional/structural genes without limiting our investigations to particular candidate genes. While potentially originating through simple changes in patterning, polymorphic polyps in *Hydractinia* are the result of differentially expressed functional, histological, and patterning genes. The DE genes identified in our study provide a starting point for future investigations of the developmental patterning and functional differences that are displayed in the different polyp types that confer a division of labor within a colony of *H. symbiolongicarpus*.

## Methods

### Animal care

Colonies of *H. symbiolongicarpus* encrusting gastropod shells occupied by the hermit crab *Pagurus longicarpus* were purchased from Marine Biological Laboratories (Woods Hole MA). Some colonies of *H. symbiolongicarpus* were surgically explanted onto microscope slides, placed in slide racks kept in seawater (REEF CRYSTALS, Aquarium Systems) aquaria, maintained at 21°C, and fed 2-3-day-old nauplii of *Artemia* three times a week. *P. longicarpus* were maintained in similar conditions and fed frozen shrimp three times a week.

### Tissue collection and RNA isolation

Tissue and RNA preps were divided into two categories based on the ultimate use of the samples (transcriptome assembly or DE analyses). Gastrozooids, gonozooids, and dactylozooids were individually dissected and collected from colonies encrusting the gastropod shells inhabited by *P. longicarpus*. The fourth polyp type (tentaculozooid) was not collected due to its rare occurrence in a colony. Excised polyps were immediately flash-frozen and stored at -80°C until RNA extractions were performed. Care was taken to only include polyp tissue and to exclude tissue from the stolons and stolonal mat of the colony. In order to obtain sufficient quantities of tissue, polyps from multiple colonies were often pooled together.

RNA extractions were carried out on pooled samples of approximately 100 individuals of a single polyp type. Total RNA was isolated using the TriReagent isolation protocol (Invitrogen) followed by a DNase treatment using the TURBO DNase kit (Ambion) or performed at the University of Kansas Medical Genome Sequencing Facility (KUMC-GSF) according to standard Illumina protocols. In samples collected for transcriptome assembly, gonozooid samples were from both male and female colonies and were pooled together during RNA extraction whereas, for the gonozooid samples collected for the downstream DE analyses, males and females were kept separate from tissue collection through sequencing.

### Library construction and sequencing

RNA libraries were constructed according to the TruSeq RNA Sample Preparation Guide (Illumina) using the TruSeq RNA Sample Preparation Kit (Box A). To increase transcript discovery, libraries used for transcriptome assembly were normalized using the Evrogen duplex-specific thermostable nuclease (DSN) kit following the Illumina DSN Normalization protocol. DNA fragments with adapters ligated on both ends were PCR-enriched after DSN normalization. Three normalized libraries were constructed with an average insert size of 160 bp and subsequently barcoded, pooled, and multiplexed across three lanes of an Illumina HiSeq2000 flowcell.

For DE analyses, a total of twelve other libraries (four for each polyp type, including two male and two female gonozooid libraries) were constructed similarly, but without DSN normalization at KUMC-GSF. These samples were barcoded, pooled, and multiplexed on a single lane of an Illumina HiSeq2500 flowcell. All libraries were 100 bp paired-end and sequenced at KUMC-GSF.

### Transcriptome assembly and annotation

The workflow from sequencing through transcript annotation and differential expression analyses is shown in Figure 2. Raw reads from all three normalized libraries were filtered based on quality score and separately mapped to a set of unpublished genomic scaffolds of *H. symbiolongicarpus* using TopHat 2.0.6 [59]. TopHat alignments were assembled into transcripts using Cufflinks 2.1.1 [60], generating three separate assemblies, one for each library. These assemblies were then merged into a single assembly using the cuffmerge function from Cufflinks [60]. This assembly was then filtered by transcript size, removing assembled transcripts less than 200 bp in length. This assembly has been submitted to the NCBI Transcriptome Shotgun Assembly (TSA) database (Accession Number GAWH00000000 [61]). The raw reads have been submitted to the NCBI Sequence Read Archive (SRA; Project Number: SRX474462).

Transcripts were annotated using several different methods. Gene Ontology (GO) terms were added with Blast2GO [62, 63], using the BLASTX algorithm and a significance threshold of 1 × 10^-03^ to search against NCBI’s non-redundant (NR) protein database. Annotation names from the GO analysis represent the top BLAST hit (Additional file 2). A set of conserved eukaryotic genes was identified with CEGMA v2.4 [64] (Additional file 3). HMM (hidden markov model) protein families from the PFAM [65] and TIGR [66] databases were assigned to the amino acid translation of the most likely reading frame (identified using an open reading frame prediction tool [67]) of each transcript using HMMscan [68] under default settings. HMMscan annotations were constrained to a significance threshold of 0.01 (Additional file 2). Orthogroups were assigned to the same amino acid translations using the orthoMCL web server [69] (Additional file 2).

### Differential expression analyses

Reads from the 12 non-normalized RNASeq libraries were mapped to the transcriptome assembly using Bowtie2 2.0.2 [70]. The raw reads from these libraries have been submitted to the NCBI Sequence Read Archive (SRA; Project Number: SRX474878). Counts for transcripts for each library were extracted from the bowtie output (.sam files) using a python script that only counts reads in which both paired reads mapped to the same transcript (Additional file 11). The count data for each library was then fed through the DESeq [71] and edgeR [72] packages to assess statistically significant DE between all pairwise combinations of polyp types, including a comparison between male and female gonozooids. Both methods were used because they often give distinctly different results, with DESeq generally being more conservative in its assessment [73–75].

Given that the goal of this study was to identify differential gene expression between somatic tissues in the different polyp types, it was necessary to reduce the effect of gametogenic expression for the DE analyses. In *Hydractinia*, there are no discernable morphological differences between male and female gonozooids aside from the type of gametes present. Thus it can be assumed that any differences in expression between male and female gonozooids can be attributed to differences in gametogenesis (heterogametic expression) and need to be accounted for prior to DE analyses between polyp types.

**Figure 7:**
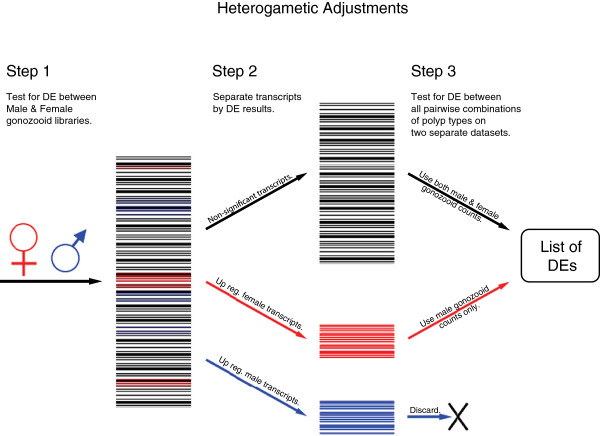
**Diagram illustrating approach taken for adjusting for gametic expression.** DE analysis was conducted between male and female gonozooid libraries (step 1), identifying significantly up- or down-regulated transcripts (step 2). Those transcripts found to be significant were excluded from the template pool and a DE analysis on those non-significant transcripts included counts from both male and female libraries (step 3 shown in black). Then, a second DE analysis was conducted on transcripts up-regulated in female gonozooids. For these comparisons, only the expression counts from male gonozooids libraries were used (step 3 shown in red). Results from the both analyses (step 3) were combined.

In an effort to distinguish between gametogenic-specific expression and expression specific to gonozooid polyp identity, several preliminary DE analyses were conducted to adjust for gametogenic expression (Figure 7). First, a DE analysis was conducted between male and female gonozooid libraries (step 1, Figure 7), identifying significantly (p_adj_ < 0.05) up- or down-regulated transcripts (step 2, Figure 7). Second, transcripts found to be significant were excluded from the template pool; a DE analysis was then performed on non-significant transcripts that included counts from both male and female libraries (step 3 shown in black, Figure 7). However, this analysis excluded maternal transcripts that could also play a role in somatic morphogenesis. To include these maternal transcripts, a second DE analysis was conducted on transcripts up-regulated in female gonozooids (putative maternal transcripts). For these comparisons, only the expression counts from the male gonozooid libraries were used (step 3 shown in red, Figure 7). In this approach, expression patterns consistent with developmental patterning and functional specialization were less likely obscured by the expression of genes specific to gametogenesis in the DE analyses. Results from both analyses (step 3, Figure 7) were combined.

These DE analyses produced a list of DE transcripts specific to one or more of the three pairwise comparisons made between the different libraries, but not ones truly specific to a certain polyp type. In order to identify these polyp-specific transcripts, only transcripts significantly up- or down-regulated (p_adj_ < 0.05) in a particular polyp when compared to either of the other two polyp types (must be significant in only two of the three pairwise comparisons) from both edgeR and DESeq were considered polyp-specific. Figure 5 is a Venn diagram that lists the number of transcripts that meet these requirements (Additional file 4).

### Probe Synthesis and *in situ* hybridization

Several polyp-specific transcripts identified during the DE analyses were selected for confirmation and further investigation with whole mount *in situ* hybridization (ISH) experiments (Table 3). Sequences for these transcripts were identified in the assembly, amplified from cDNA, cloned using the Invitrogen TOPO-TA Cloning Kit, and anti-DIG labeled riboprobes were synthesized from clones using the Invitrogen T7/T3 Megascript kit. ISH of these transcripts were performed following methods from Nawrocki & Cartwright [76].

### Molecular phylogenetic analyses

Several gene trees were constructed of select gene families, including homeodomains, myosins, toxins, and astacins (Additional file 6; Additional file 7; Additional file 8; Additional file 9). Cnidarian sequences belonging to families of interest were mined from the nr NCBI database and aligned using Mafft [77]. Depending on the family, either the L-insi or E-insi alignment algorithm was used. Only polyp-specific *H. symbiolongicarpus* sequences annotated with these families were included in the alignments. Maximum likelihood estimates of the molecular phylogenies of these gene families were then produced using RAxML [78] on the CIRPES portal [79] using the rapid bootstrapping (-f a) algorithm with 1000 bootstrap replicates under the PROTGAMMA + WAG model.

## Electronic supplementary material

Additional file 1: **Histogram of the size distribution of assembled transcripts.** This does not include transcripts that were removed because they were < 200 bp in length. Inset table displays assembly numbers and size statistics before and after filtering out the <200 bp transcripts. (PDF 145 KB)

Additional file 2: **Blast2GO, HMMscan, and orthoMCL annotations of all transcripts.** (CSV 10 MB)

Additional file 3: **CEGMA output.** (ZIP 326 KB)

Additional file 4: **All polyp-specific transcripts.** This list includes the assembly sequence ID, top BLASTX hit, number of gene ontology IDs, top HMM protein domain, polyp specificity, top significance threshold, and transcript sequence. Polyp specificity is defined in two separate columns, ‘Polyp’ and ‘Direction’ (example: ‘Gono, DOWN, .05,’ would be a transcript that is significantly down regulated in the gonozooid when compared to the other two polyps at a significance level between .01 and .05). (CSV 9 MB)

Additional file 5: **List of polyp-specific DEs discussed in** **Results and discussion** **section.** This table is a subset of Additional file 4 and contains all the same information, but with BS support and the accession number of the closest cnidarian ortholog, if molecular phylogenetic analyses performed. In cases where phylogenetic analyses recovered well supported sister relationships between two monophyloetic clades with no clear one to one orthologous relationships, a single accession number was selected from the non-*H. symbiolongicarpus monophyly*. List is divided up by color according to the subheading in the Results and discussion section they are discussed in: Blue – ‘Gametogenic expression’; Yellow – ‘Homeodomains’; Pink – ‘Myosins’; Green – ‘Toxins’; Orange – ‘Astacins’. (*) Marks transcripts with whole mount in situ hybridizations data in this study. (XLSX 152 KB)

Additional file 6: **Cnidarian homeodomain gene tree.** Molecular phylogeny of cnidarian homeodomains sampled from GenBank’s nr database. Accession numbers are appended to the ends of the tip labels. Only polyp-specific homeodomains from *H. symbiolongicarpus* (highlighted in red) were included in the analysis. Fasta and alignment file available upon request. (PDF 12 KB)

Additional file 7: **Cnidarian myosin gene tree.** Molecular phylogeny of cnidarian myosins sampled from GenBank’s nr database. Accession numbers are appended to the ends of the tip labels. Only polyp-specific homeodomains from *H. symbiolongicarpus* (highlighted in red) were included in the analysis. Fasta and alignment file available upon request. (PDF 4 KB)

Additional file 8: **Cnidarian toxin gene tree.** Molecular phylogeny of cnidarian toxins sampled from GenBank’s nr database. Accession numbers are appended to the ends of the tip labels. Only polyp-specific homeodomains from *H. symbiolongicarpus* (highlighted in red) were included in the analysis. Fasta and alignment file available upon request. (PDF 4 KB)

Additional file 9: **Cnidarian astacin gene tree.** Molecular phylogeny of cnidarian astacins sampled from GenBank’s nr database. Accession numbers are appended to the ends of the tip labels. Only polyp-specific astacins from *H. symbiolongicarpus* (highlighted in red) were included in the analysis. Fasta and alignment file available upon request. (PDF 5 KB)

Additional file 10: **In situ hybridization (higher magnification) of toxins.** A. toxin_5320. B. toxin_3875. sp = spumeous cells; ns = nematocyst; nc = nematocyte. (PDF 5 MB)

Additional file 11: **Python script for extracting count data from .sam files.** Save as counts-paired.py. *Use: python counts-paired.py infile.sam.* (CSV 2 KB)

## Abbreviations

DE: Differential expression or differentially expressed
NGS: Next generation sequencing
BP: Base pair
ISH: Whole mount *in situ* hybridization
DSN: Duplex-Specific thermostable nuclease
GO: Gene ontology
HMM: Hidden markov model
ORF: Open reading frame.
