# Supplementary figures and images for: Differential gene expression between functionally specialized polyps of the colonial hydrozoan Hydractinia symbiolongicarpus (Phylum Cnidaria)
Source: BMC Genomics. 2014 May 28;15(1):406. doi: 10.1186/1471-2164-15-406 (PMC4072882; doi:10.1186/1471-2164-15-406)

Size Distribution of Assembled Transcripts

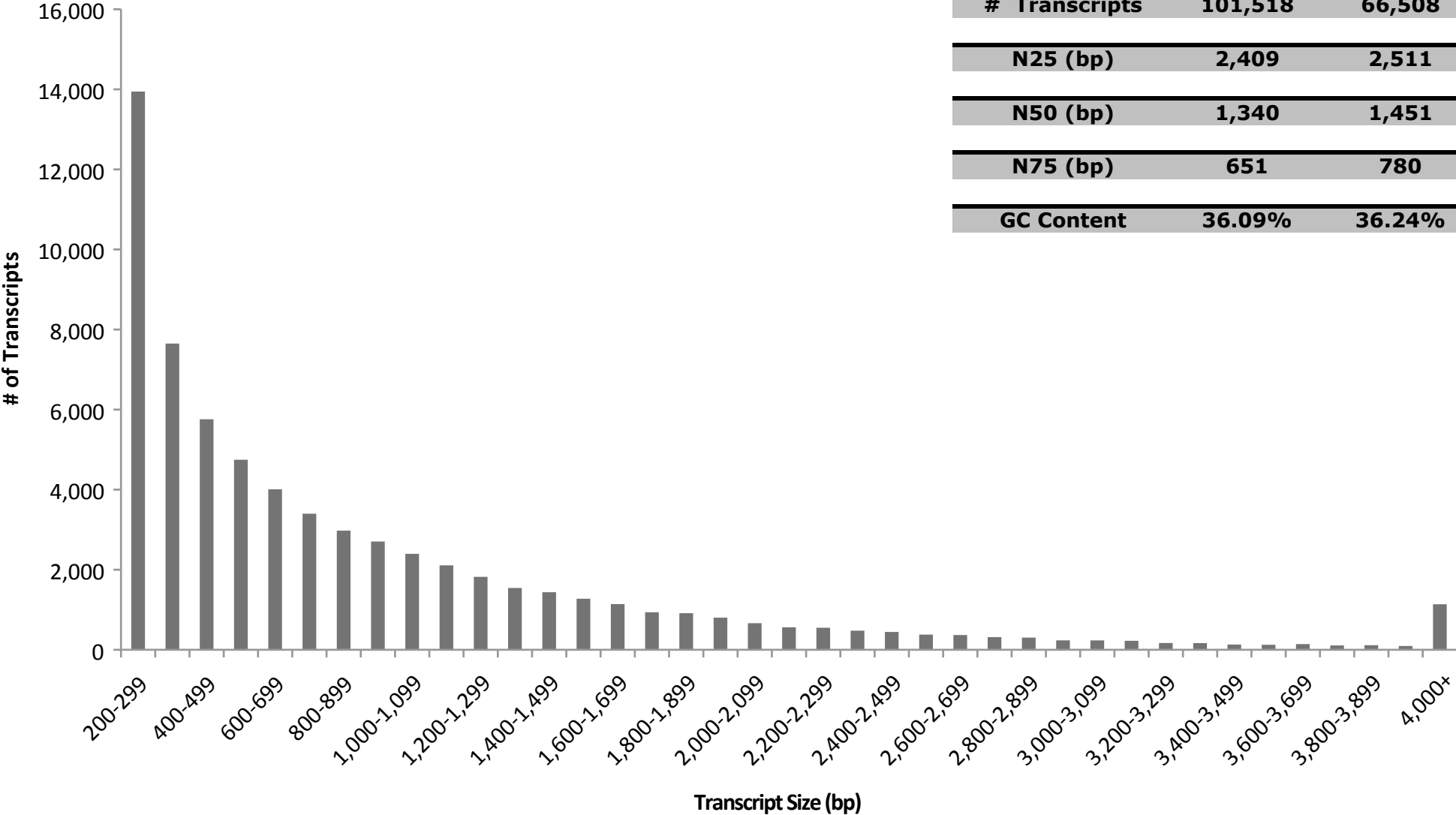

|               | All     | Filtered |
|---------------|---------|----------|
| # Transcripts | 101,518 | 66,508   |
| N25 (bp)      | 2,409   | 2,511    |
| N50 (bp)      | 1,340   | 1,451    |
| N75 (bp)      | 651     | 780      |
| GC Content    | 36.09%  | 36.24%   |

Supplement: Supplementary file 1 — Additional file 1: Histogram of the size distribution of assembled transcripts. This does not include transcripts that were removed because they were < 200 bp in length. Inset table displays assembly numbers and size statistics before and after filtering out the <200 bp transcripts. (PDF 145 KB) [file 12864_2014_6153_MOESM1_ESM.pdf]

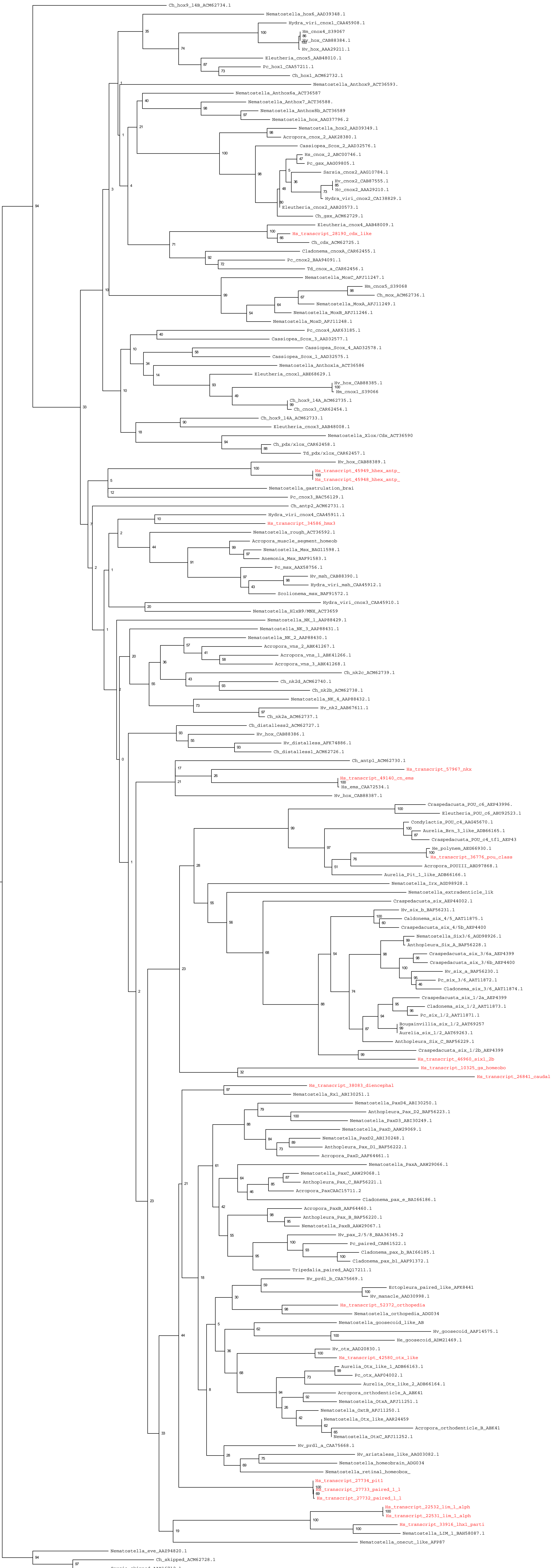

Supplement: Supplementary file 6 — Additional file 6: Cnidarian homeodomain gene tree. Molecular phylogeny of cnidarian homeodomains sampled from GenBank’s nr database. Accession numbers are appended to the ends of the tip labels. Only polyp-specific homeodomains from H. symbiolongicarpus (highlighted in red) were included in the analysis. Fasta and alignment file available upon request. (PDF 12 KB) [file 12864_2014_6153_MOESM6_ESM.pdf]

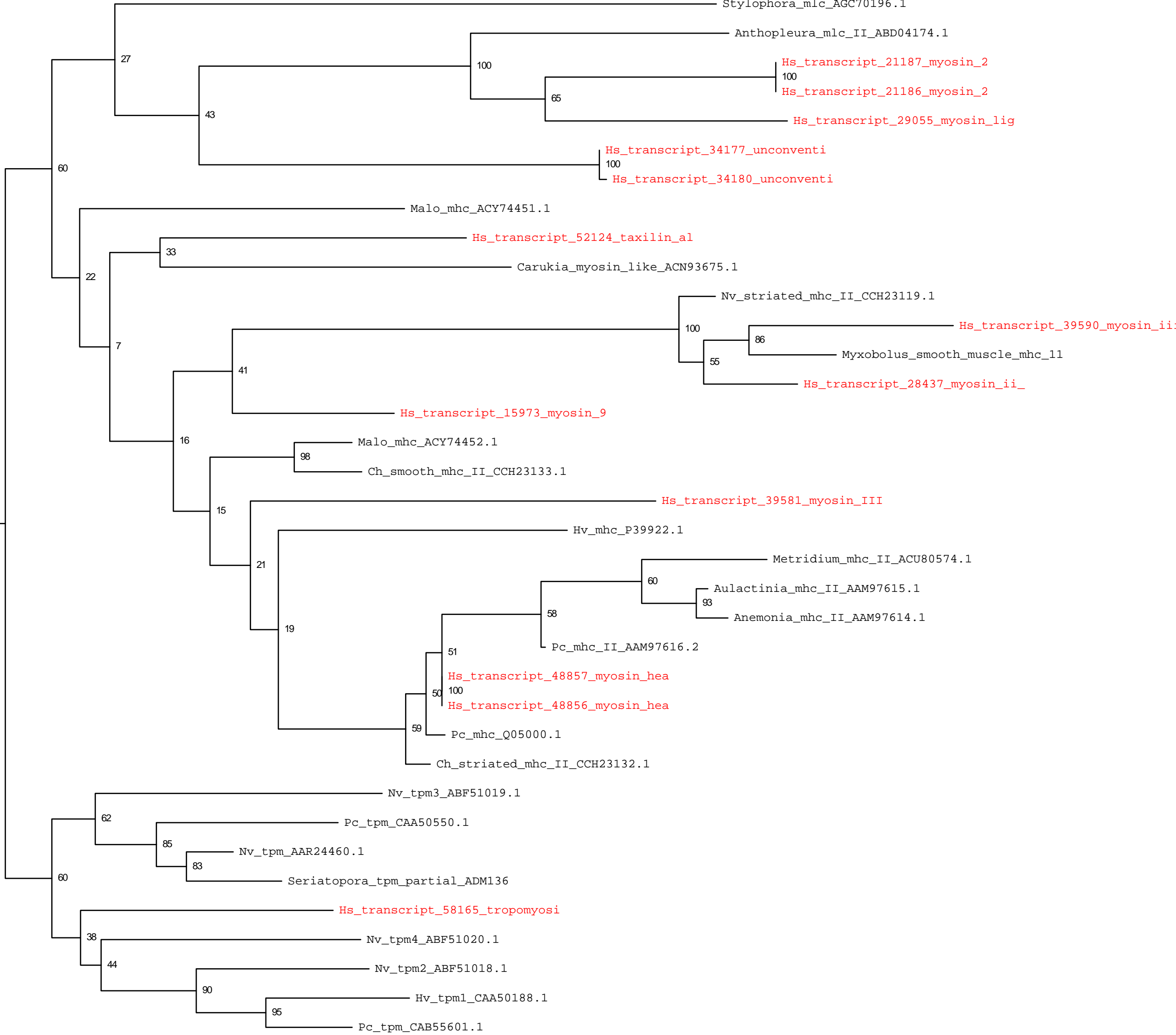

0.4

Supplement: Supplementary file 7 — Additional file 7: Cnidarian myosin gene tree. Molecular phylogeny of cnidarian myosins sampled from GenBank’s nr database. Accession numbers are appended to the ends of the tip labels. Only polyp-specific homeodomains from H. symbiolongicarpus (highlighted in red) were included in the analysis. Fasta and alignment file available upon request. (PDF 4 KB) [file 12864_2014_6153_MOESM7_ESM.pdf]

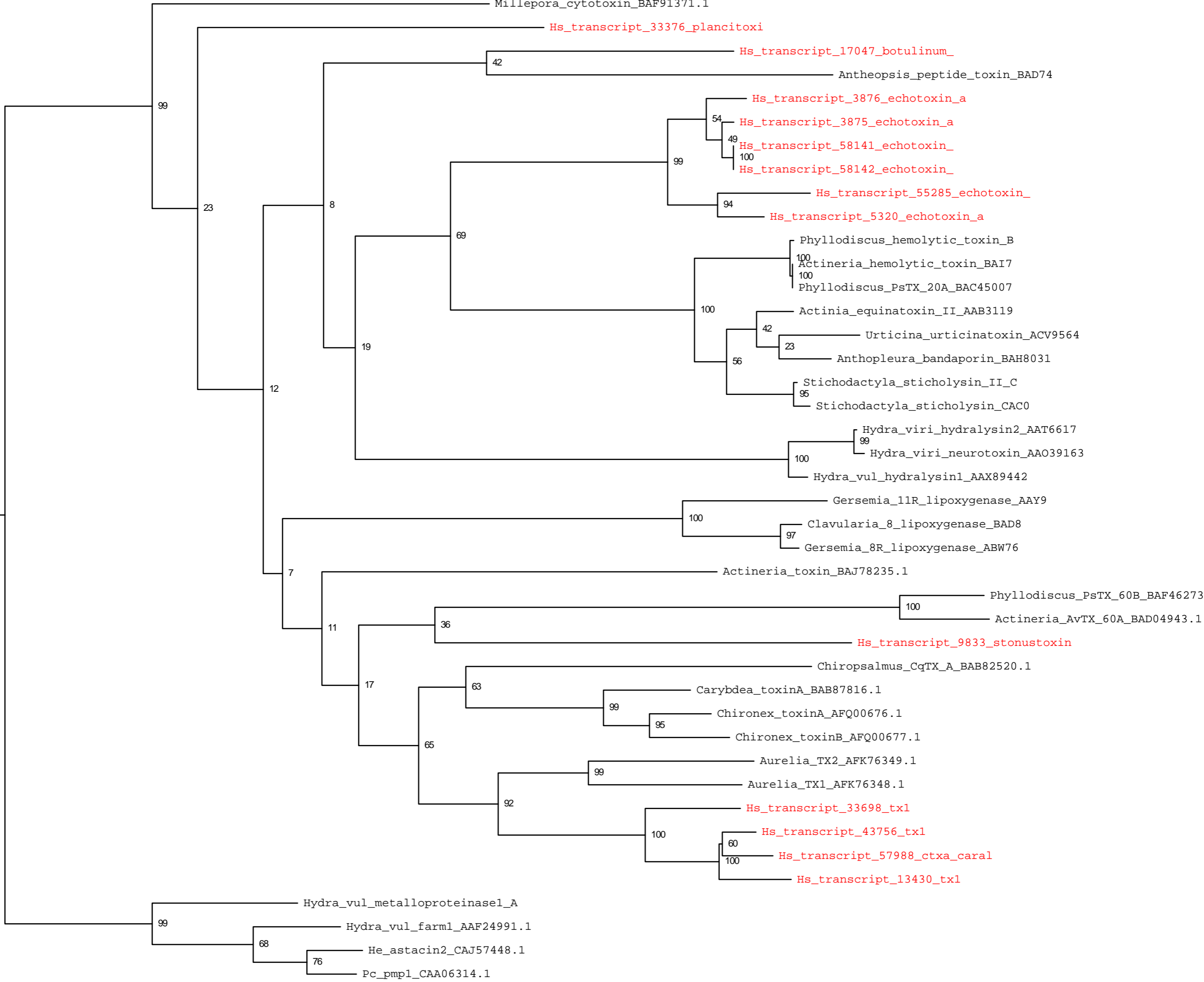

0.4

Supplement: Supplementary file 8 — Additional file 8: Cnidarian toxin gene tree. Molecular phylogeny of cnidarian toxins sampled from GenBank’s nr database. Accession numbers are appended to the ends of the tip labels. Only polyp-specific homeodomains from H. symbiolongicarpus (highlighted in red) were included in the analysis. Fasta and alignment file available upon request. (PDF 4 KB) [file 12864_2014_6153_MOESM8_ESM.pdf]

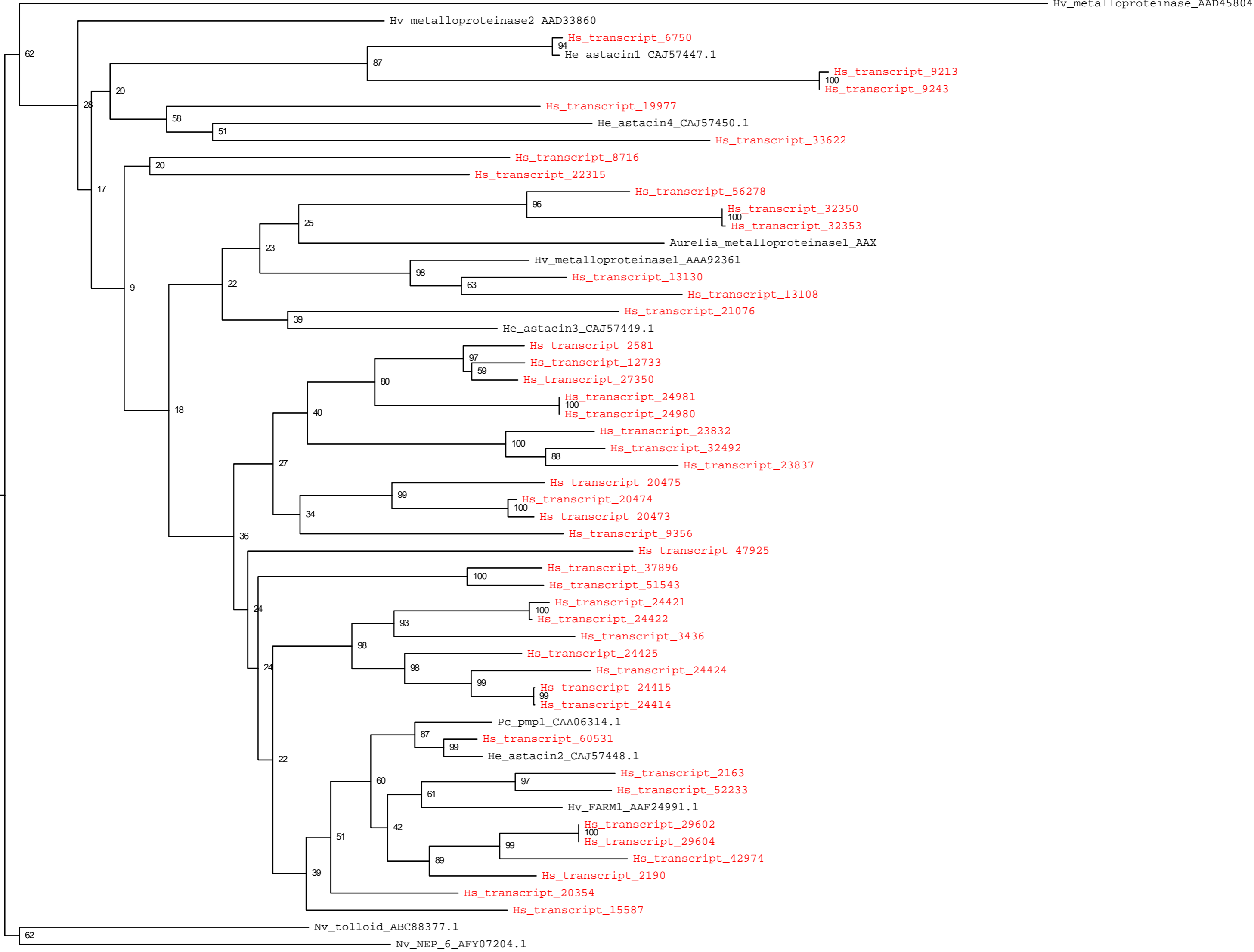

Hv\_metalloproteinase\_AAD45804.

0.4

Supplement: Supplementary file 9 — Additional file 9: Cnidarian astacin gene tree. Molecular phylogeny of cnidarian astacins sampled from GenBank’s nr database. Accession numbers are appended to the ends of the tip labels. Only polyp-specific astacins from H. symbiolongicarpus (highlighted in red) were included in the analysis. Fasta and alignment file available upon request. (PDF 5 KB) [file 12864_2014_6153_MOESM9_ESM.pdf]

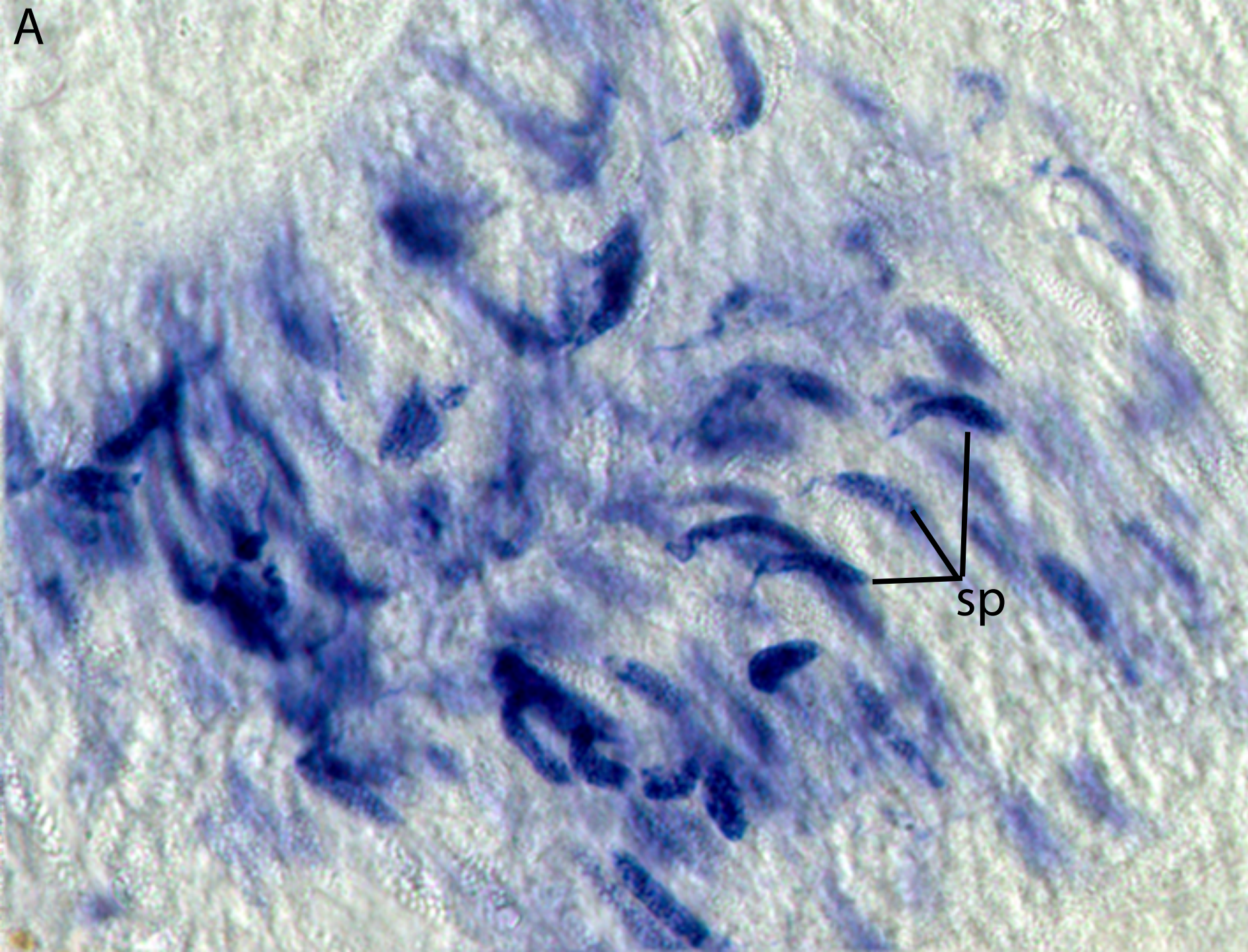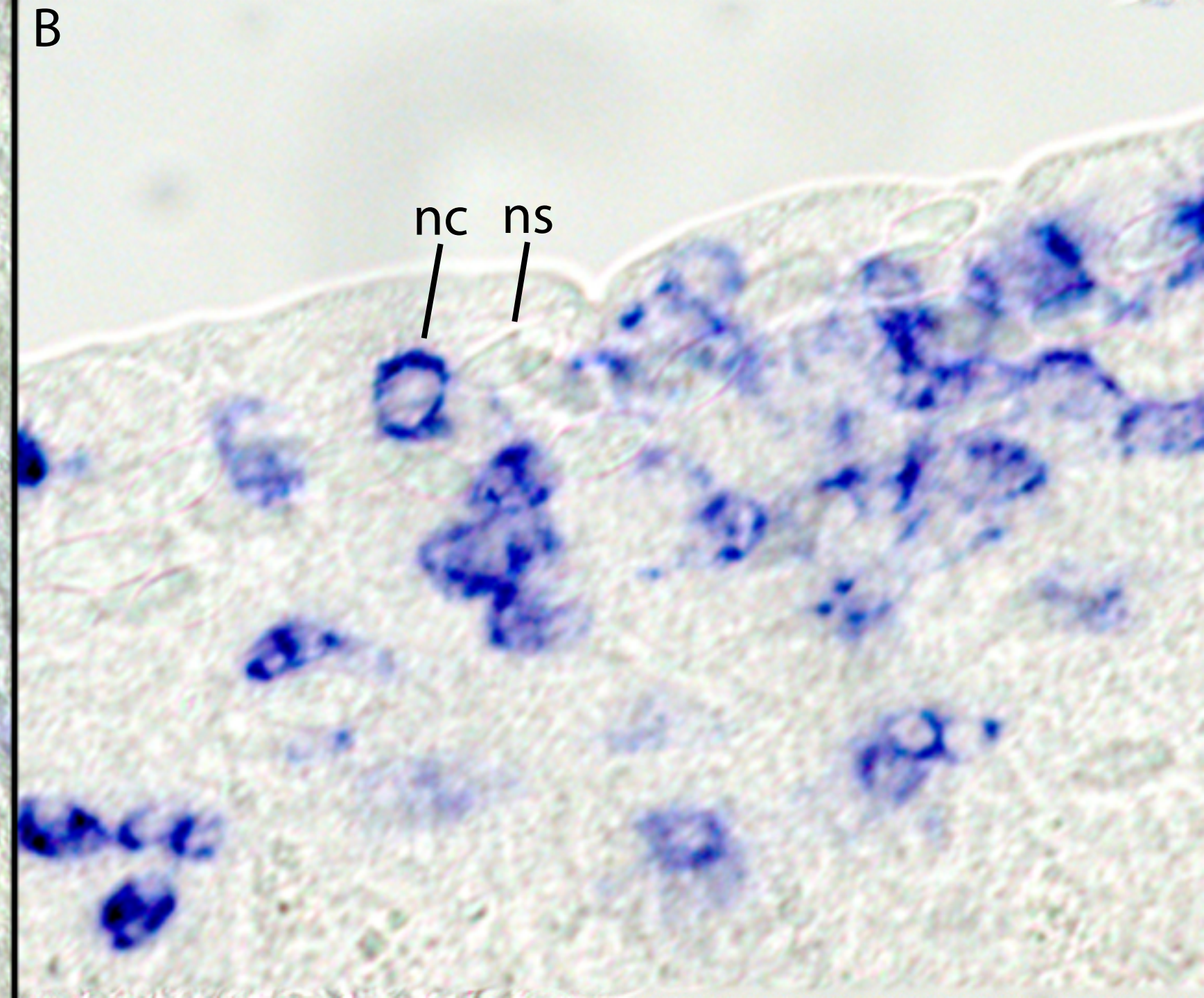

Supplement: Supplementary file 10 — Additional file 10: In situ hybridization (higher magnification) of toxins. A. toxin_5320. B. toxin_3875. sp = spumeous cells; ns = nematocyst; nc = nematocyte. (PDF 5 MB) [file 12864_2014_6153_MOESM10_ESM.pdf]
